# Inverse design of compact silicon photonic waveguide reflectors and their application for Fabry–Perot resonators

**DOI:** 10.1515/nanoph-2024-0017

**Published:** 2024-04-16

**Authors:** Yonghan Kim, Sung-Hoon Hong

**Affiliations:** Photonic and Wireless Devices Research Division, Terrestrial and Non-Terrestrial Integrated Telecommunications Research Laboratory, 65678 Electronics and Telecommunications Research Institute (ETRI), Daejeon, 34129, Republic of Korea; Material and Component Research Division, Superintelligence creative Research Laboratory, 65678 Electronics and Telecommunications Research Institute (ETRI), Daejeon, 34129, Republic of Korea

**Keywords:** inverse design, photonic waveguide reflector, Fabry–Perot resonator

## Abstract

Silicon photonic waveguide resonators, such as microring resonators, photonic crystal waveguide cavities, and Fabry–Perot resonators based on the distributed Bragg reflectors, are key device components for silicon-based photonic integrated circuits (Si-PIC). For the Si-PIC with high integration density, the device footprints of the conventional photonic waveguide resonators need to be more compact. Inverse design, which is operated by the design expectation and different from the conventional design methods, has been investigated for reducing the photonic device components nowadays. In this paper, we inversely designed the silicon photonic waveguide reflectors for two target wavelengths: one is 1310 nm and the other is 1550 nm. The silicon photonic waveguide reflectors have reflectance of 0.99993 and 0.9955 for the wavelength of 1310 nm and 1550 nm each with 5-μm-long reflectors. Also, we theoretically investigated Fabry–Perot resonators based on the inversely designed photonic waveguide reflectors. *Q* factors of the Fabry–Perot resonators have been calculated to be 1.3 × 10^5^ for the wavelength of 1310 nm and 2583 for the wavelength of 1550 nm. We have expected that the inversely designed photonic waveguide reflectors and their applications for the Fabry–Perot resonators can be utilized for compact passive/active device components such as wavelength filters, modulators, and external cavity lasers.

## 1 Introduction

For few decades, silicon photonics has been spotlighted due to its CMOS-compatible fabrication processes and realization of the large-scale photonic integrated circuits (PICs) with high speed and low energy consumption [1]. As silicon photonics have been established, integrations of the optical waveguide resonators, which store light energy in small cavities made of silicon optical waveguides, in the silicon-based PICs (Si-PICs) are widely performed in a variety of ways. For instance, there are several types of the optical resonators for silicon photonics including microring resonators (MRRs) [2], [3], [4], [5], photonic crystal (PhC) waveguide cavities [6], [7], [8], and Fabry–Perot (FP) resonator based on distributed Bragg reflectors (DBRs) [9], [10]. Those optical resonators have been utilized for many photonic device components including wavelength filters [11], [12], [13], [14], optical modulators [15], [16], [17], [18], sensors [19], [20], [21], and external cavity lasers (ECLs) [22], [23], [24], [25] and so on. As the importance of PICs with high density has been emerged, the device footprints of the optical resonators need to be reduced. However, reducing the sizes of the optical resonators with conventional design methods has a limitation. In case of the MRRs, the bending radius of the silicon-based MRR is limited to few μm due to radiation losses [2]. And, placement of the microring, which is naturally next to the bus waveguide for the coupling light, would reduce the integration density of the PICs. In cases of the PhC waveguide cavities, unlikely to the MRRs, the device footprints are much smaller since the cavity mode of PhC is determined by the photonic bandgap [7]. However, in order to couple the light to PhC waveguide cavities, special waveguide structures should be considered in order to overcome the slow light effect of the photonic bandgap [26]. And, in case of the DBRs, the number of gratings should be sufficient to have enough reflectivity such that total length of DBR is about tens μm [10]. If there are any alternative designs for reducing the sizes of the photonic waveguide resonators, the density of the PICs would be able to be enhanced.

Inverse design, which is a design methodology that is operated by the design expectation rather than design equations or theories, which are essential for conventional designs, would be a candidate for reducing the sizes of the photonic waveguide device components. The inverse designs for the photonic devices including power splitters [27], [28], [29], mode converters [30], [31], grating couplers [32], and single photon sources [33] have been investigated. Not only reducing the device footprints of the photonic devices but simplifying the design steps of the photonic devices is the most powerful advantage of the inverse design for photonic devices compared to other conventional design methods. Inverse design for the photonic devices enables unique device performances. For example, in case of the power splitters, the conventional method of the power splitting is using Y-branch [34] or multimode-interference (MMI)-based 3 dB splitter [35]. Those conventional power splitters are used for splitting the power into a half for each waveguide port. If we want to split the power into larger numbers of waveguide ports, very complex designs should be considered and the total device footprints should be increased. However, the inversely designed power splitter shows the power split ratio of 1/3 with a compact device footprint which is just 3.8 μm × 2.5 μm [27]. Also, other inversely designed photonic devices have much more compact sizes compared to the conventional photonic devices. For example, in case of the grating coupler, the size of the conventional grating coupler is about 100 μm. However, in case of the inversely designed grating coupler, the size of the device is just 8 μm [32]. It is about 12 times smaller than the sizes of the conventional grating couplers.

In this paper, we theoretically investigate a photonic waveguide reflector by the inverse design method for integrating Fabry–Perot resonators with the inversely designed photonic waveguide reflectors. We have inversely designed the photonic waveguide reflectors, which have near perfect reflectance with just 5-μm-long devices for the target wavelengths of 1310 nm and 1550 nm. Also, we have expected that this photonic waveguide reflector can be an alternative design for the conventional DBR device and investigated a potential of the inversely designed photonic waveguide reflector for the photonic waveguide Fabry–Perot resonators.

## 2 Inverse design of Si photonic waveguide reflectors

### 2.1 Simulation schematic

**Figure 1:**
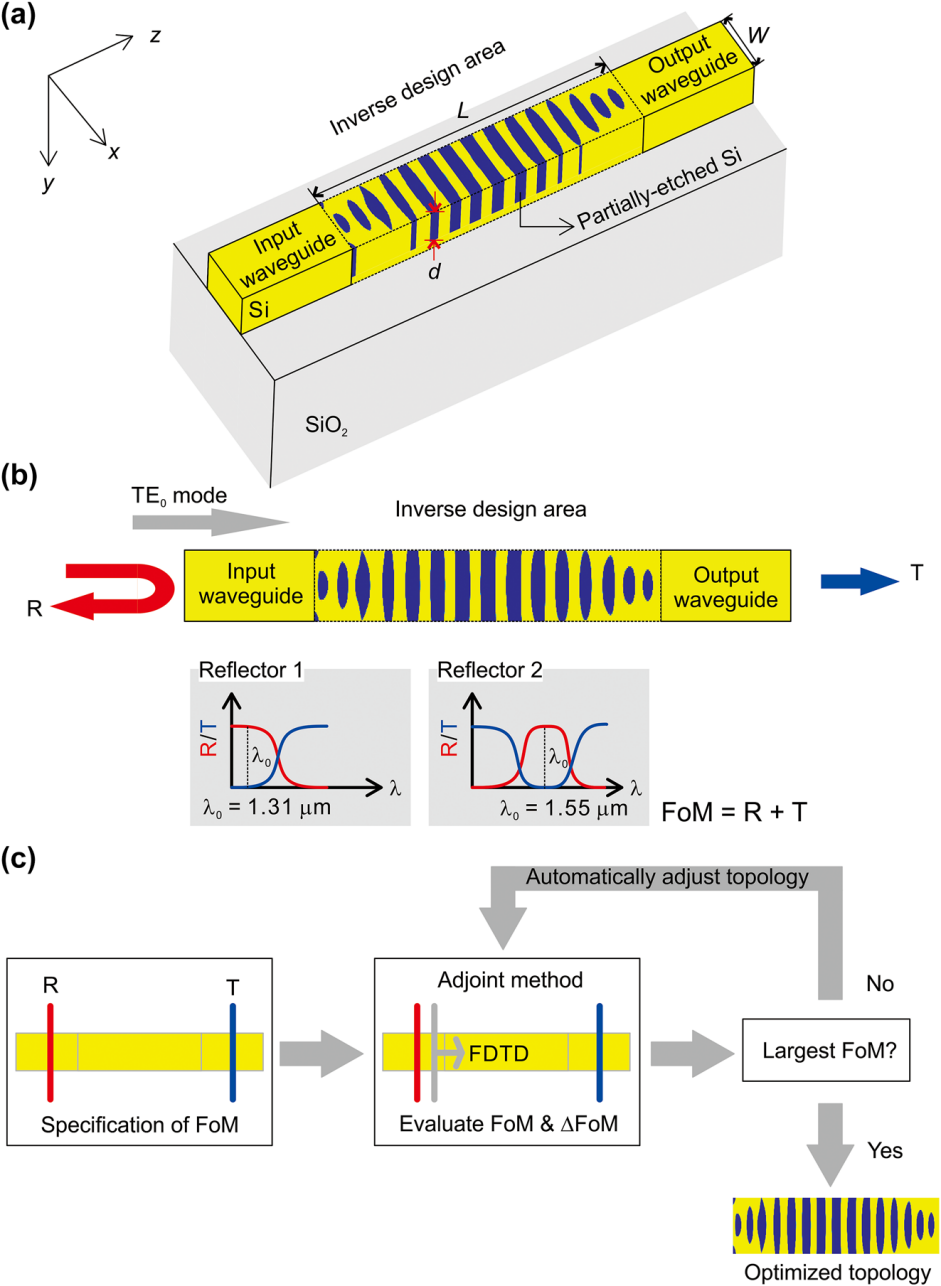
Simulation schematic of the inverse design of Si photonic waveguide reflector. (a) Isometric view of the simulation schematic. (b) Description of the concept of the inverse design for the Si photonic waveguide reflectors. (c) A flowchart of the inverse design.

In order to simulate the inverse design of the photonic waveguide reflector, we conducted the finite-difference time-domain (FDTD) simulation (Ansys Lumerical) with Python programming language. Figure 1 describes the simulation schematic for the inverse design. The simulation has three regions. There are input waveguide, inverse design area, and output waveguide. The input and output waveguide are silicon (Si) straight waveguides on a 2-μm-thick silicon dioxide (SiO_2_) substrate. We assume the temperature for the simulation of the inversely designed photonic waveguide reflectors is 25 °C. The width and height of the waveguide is set to 0.45 μm and 0.22 μm each. In the inverse design area, we assume that partially etched silicon pattern in the straight waveguide. The etch depth of Si (*d*) is one of inverse design parameters. The geometric topology of the partially etched pattern is determined by the inverse design. While performing the inverse design, we have estimated the reflectance at a target wavelength band and the transmittance at a wavelength range outside the target wavelength band. The quasi-fundamental transverse electric (TE_0_) mode is launched at the input waveguide as the light source, and the reflectance of the device is measured behind the position of the light source and the transmittance of the device is at the output waveguide. In order to simulate the inverse design area, the inverse design area is divided into small nodes, which have a dimension of d*x* × d*y*, and they are set to 10 nm. So, total number of nodes is determined to be *L*/d*x* × *W*/d*y*. *L* is the length of the inverse design area and *W* is the width of the inverse design area. *W* is set to 0.45 μm, which is identical to the width of the waveguide, and *L* is another inverse design parameter. Figure 1(c) shows a flowchart of the inverse design for the Si photonic waveguide reflector. Initially target reflectance and transmittance is specified as a Figure-of-Merit (FoM). Then, the FDTD simulation is conducted for evaluating FoM and gradient of FoM (ΔFoM). After that if FoM is the largest, the inverse design would be done and makes optimized topology of the device, or if not, it returns to automatically modified topology and re-evaluates FoM and ΔFoM until the FoM reaches the largest value. In order to fabricate this optimized topology on the Si waveguide surface, the possible fabrication procedure would be followed. First, the Si photonic waveguide is fabricated on a silicon-on-insulator (SOI) wafer with 220-nm-thick Si layer and 2-μm-thick SiO_2_ layer by a photolithography and reactive ion etch (RIE). Then, an electron-beam (e-beam) lithography is used for refining the inversely designed pattern on the waveguide with an e-beam resist, which has a high selectivity for the Si etching process. After the e-beam lithography, another RIE process is conducted for the partial etch of the inverse design. Finally, remained e-beam resist would be removed by O_2_ plasma.

### 2.2 Figure-of-merit of the Si photonic waveguide reflectors

In this paper, we have inversely designed two photonic waveguide reflectors: one has a target wavelength of 1310 nm (Reflector 1) and the other has a target wavelength of 1550 nm (Reflector 2). The optical bandwidth for the reflection of each reflector is set to 100 nm. For the inverse design, we used an adjoint-based optimization method for the inverse design of the photonic waveguide reflectors [36]. We set the figure-of-merits (FoMs) of the inverse design for the photonic waveguide reflectors as a summation of the reflectance at the target wavelength band and the transmittance at a wavelength range outside the target wavelength band simultaneously. For example, in case of the Reflector 1, which has a target wavelength band from 1260 nm to 1360 nm, it is inversely designed to achieve a maximum reflectance at a wavelength range from 1260 nm to 1360 nm and a maximum transmittance except that wavelength band. In order to estimate the reflectance and the transmittance of the device, we calculate the mode overlap between the TE_0_ mode and the electromagnetic fields at the positions where the reflectance and the transmittance are estimated and it is explained in Equations (1). In Equations (1), ***E***_***R***_ (***E***_***T***_) and ***H***_***R***_ (***H***_***T***_) are the electric field and the magnetic field calculated at the position for reflectance (transmittance). ***E***_**0**_ and ***H***_**0**_ are the electric field and the magnetic field of the TE_0_ mode.

(1a) $$R\left(\lambda \right)=\frac{1}{8}\frac{{\left|\int E_{R}\left(\lambda \right)\times \bar{H_{0}\left(\lambda \right)}\cdot dS+\int \bar{E_{0}\left(\lambda \right)}\times H_{R}\left(\lambda \right)\cdot dS\right|}^{2}}{\int \text{Re}\left(E_{0}\left(\lambda \right)\times \bar{H_{0}\left(\lambda \right)}\right)\cdot dS}$$

(1b) $$T\left(\lambda \right)=\frac{1}{8}\frac{{\left|\int E_{T}\left(\lambda \right)\times \bar{H_{0}\left(\lambda \right)}\cdot dS+\int \bar{E_{0}\left(\lambda \right)}\times H_{T}\left(\lambda \right)\cdot dS\right|}^{2}}{\int \text{Re}\left(E_{0}\left(\lambda \right)\times \bar{H_{0}\left(\lambda \right)}\right)\cdot dS}$$

Equation (1) is just for a single wavelength. In order to consider the optical bandwidth, we estimated wavelength-averaged reflectance and transmittance by Equations (2).

(2a) $$R=\frac{1}{\lambda_{2}-\lambda_{1}}\overset{\lambda_{2}}{\underset{\lambda_{1}}{\int }}R\left(\lambda \right)\text{d}\lambda$$

(2b) $$T=\frac{1}{\lambda_{\max}-\lambda_{2}}\overset{\lambda_{\max}}{\underset{\lambda_{2}}{\int }}T\left(\lambda \right)\text{d}\lambda +\frac{1}{\lambda_{1}-\lambda_{\min}}\overset{\lambda_{1}}{\underset{\lambda_{\min}}{\int }}T\left(\lambda \right)\text{d}\lambda$$

In Equations (2), the wavelength range from *λ*_1_ to *λ*_2_ corresponds to an optical bandwidth, which is set by inverse design condition, and *λ*_max_ and *λ*_min_ are maximum and minimum wavelengths in the simulation. So, the final FoM of the device is calculated to summation of the wavelength-averaged reflectance and transmittance by Equations (2).

### 2.3 Simulation results of inverse designs of Si photonic waveguide reflectors

**Figure 2:**
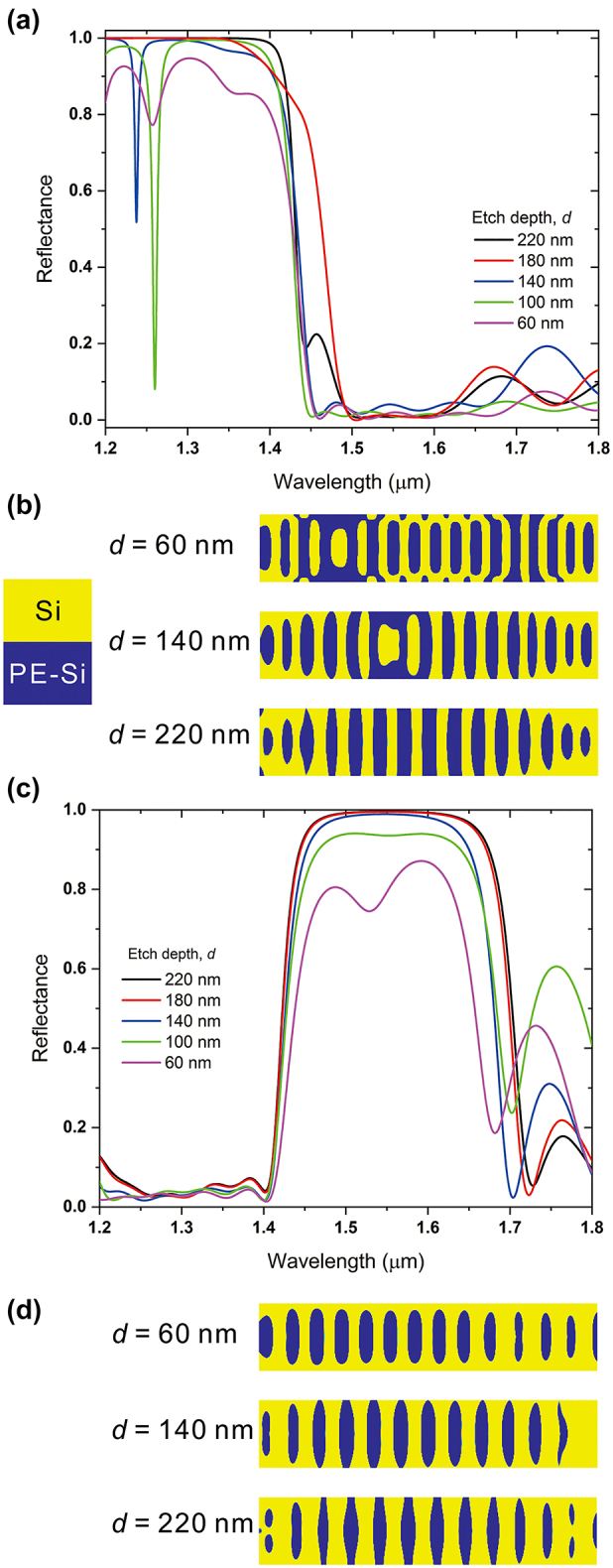
Effect of etch depth of Si on the inversely designed reflector. (a) Reflectance spectra of the Reflector 1, which has a target wavelength of 1310 nm. (b) Calculated surface topology of three values of etch depths of Reflector 1. Yellow color indicates the region occupied by Si and blue color indicates partially etched Si region (PE-Si). (c) Reflectance spectra of the Reflector 2, which has a target wavelength of 1550 nm. (d) Calculated surface topology of three values of etch depths of Reflector 2.

We simulated the inverse designs of the photonic waveguide reflectors with inverse design parameters including etch depth d and length of the inverse design area L. First, we investigated the effects of etch depths on the photonic waveguide reflectors and they are shown in Figure 2 for both Reflector 1 (Figure 2(a)) and Reflector 2 (Figure 2(c)). The surface topologies of each Reflector with different values of *d* are shown in Figure 2(b)–(d). When *d* is 220 nm, which indicates fully etched Si, Reflector 1 with *L* = 5 μm shows the reflectance of 0.99993, which is almost perfect reflection, and transmittance of 5 × 10^−5^ at the target wavelength of 1310 nm. However, when *d* is reduced to 60 nm, the reflectance of the Reflector 1 is reduced to 0.945 and the transmittance of the Reflector 1 is increased to 0.055 simultaneously at the target wavelength. In case of the Reflector 2, for the *d* = 220 nm, the reflectance and transmittance of the Reflector 2 are 0.9955 and 0.0045 each at the target wavelength of 1550 nm. Similar to Reflector 1, the reflectance of the Reflector 2 is decreased to 0.794 and the transmittance is increased to 0.207 when *d* is decreased to 60 nm. The larger etch depth indicates the larger refractive indices difference between Si and partially etched Si, which makes the stronger reflection by the designed structures.

In order to verify the advantages of the inverse design for the waveguide reflectors, we compared the Si waveguide DBRs with same etch depths with the inversely designed Si photonic waveguide reflectors in Supplementary Material. From the results in Figure S1, compared to the inversely designed reflectors, the DBRs shows lower reflectance for both target wavelength bands. In case of the target wavelength of 1310 nm, the reflectance of the DBR is calculated to be 0.9497 for *d* = 220 nm, and it is reduced to 0.728 when *d* is reduced to 60 nm. Also, in case of the target wavelength of 1550 nm, the reflectance of the DBR is estimated to 0.9491 for *d* = 220 nm, and it is reduced to 0.7052 when *d* is reduced to 60 nm. Not only the reduced reflectance but also the transmission outside the target wavelength band is increased.

**Figure 3:**
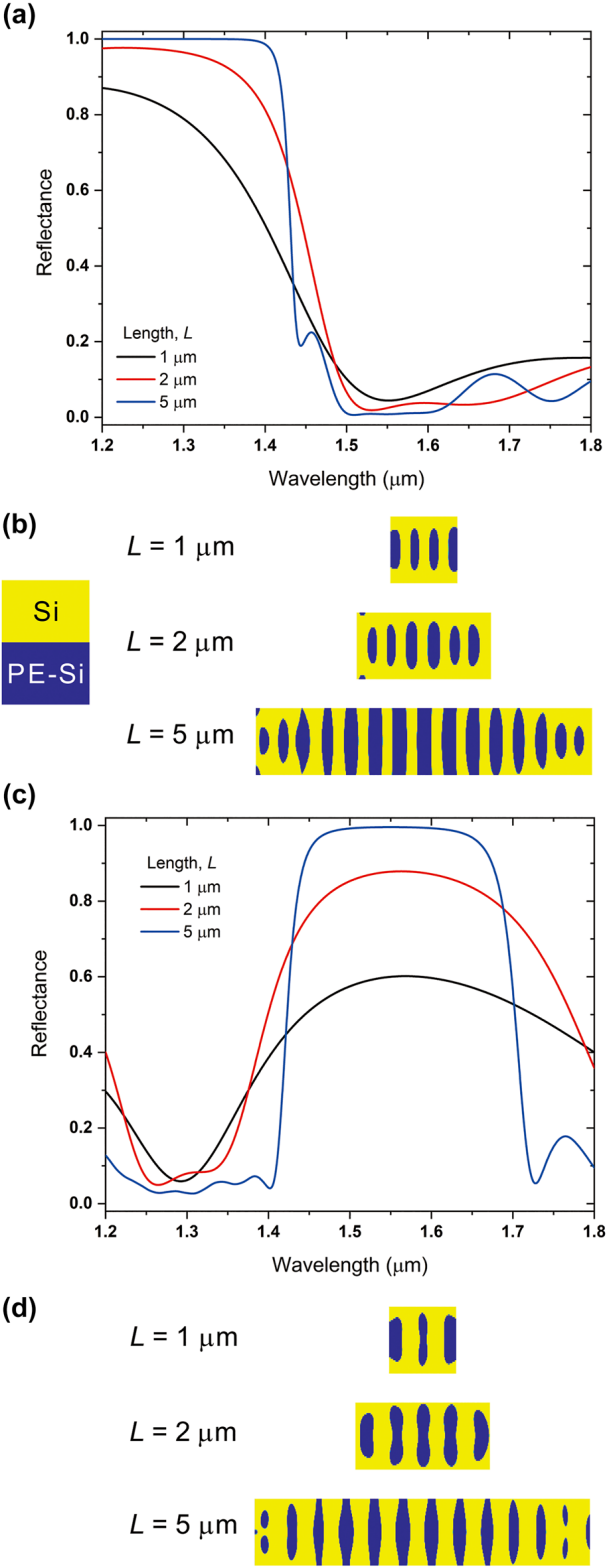
Effect of inverse design length on the inversely designed reflector. (a) Reflectance spectra of the Reflector 1, which has a target wavelength of 1310 nm. (b) Calculated surface topology of three values of etch depths of Reflector 1. Yellow color indicates the region occupied by Si and blue color indicates partially etched Si region (PE-Si). (c) Reflectance spectra of the Reflector 2, which has a target wavelength of 1550 nm. (d) Calculated surface topology of three values of etch depths of Reflector 2.

Then we investigate the effect of the length of the inverse design area on the reflectance of each Reflector. The reflectance and transmittance spectra of the Reflector 1 and Reflector 2 are shown in Figure 3(a)–(c) for the three values of L, which are 1 μm, 2 μm, and 5 μm with fixed values of *d*. For the Reflector 1, the reflectance of 5-μm-long device is 0.99993 at the wavelength of 1310 nm. When the length of the device is reduced to 2 μm, the reflectance of the device is just slightly decreased to 0.9602. However, when the length of the device is reduced to 1 μm, the reflectance of the device is reduced to 0.7727 as well. In case of the Reflector 2, the reflectance of the 5-μm-long device is 0.9955 at the wavelength of 1550 nm. And, when the length of the device is decreased to 2 μm, the reflectance of the device is 0.8777. However, when the length of the device is reduced to 1 μm, the reflectance of the device is 0.5998 at the wavelength of 1550 nm, which is very similar to the Reflector 1.

Also, we compared the Si waveguide DBRs with different DBR lengths for verification of the advantages of the inverse design for the waveguide reflectors and the results of the DBRs in Supplementary Material, Figure S2. In Figure S2, 5-μm-long DBRs for both target wavelengths show good reflection performances. However, when the length of the DBRs is decreased to 2 μm, the reflection performance of the DBR is deteriorated. For the target wavelength of 1310 nm, the reflectance of DBR is 0.804 contrasting with the reflectance of 0.9602 for the inversely designed reflector. Also, for the target wavelength of 1550 nm, the reflectance of DBR is just 0.760, which is 1.15 times lower than the inversely designed reflector.

**Figure 4:**
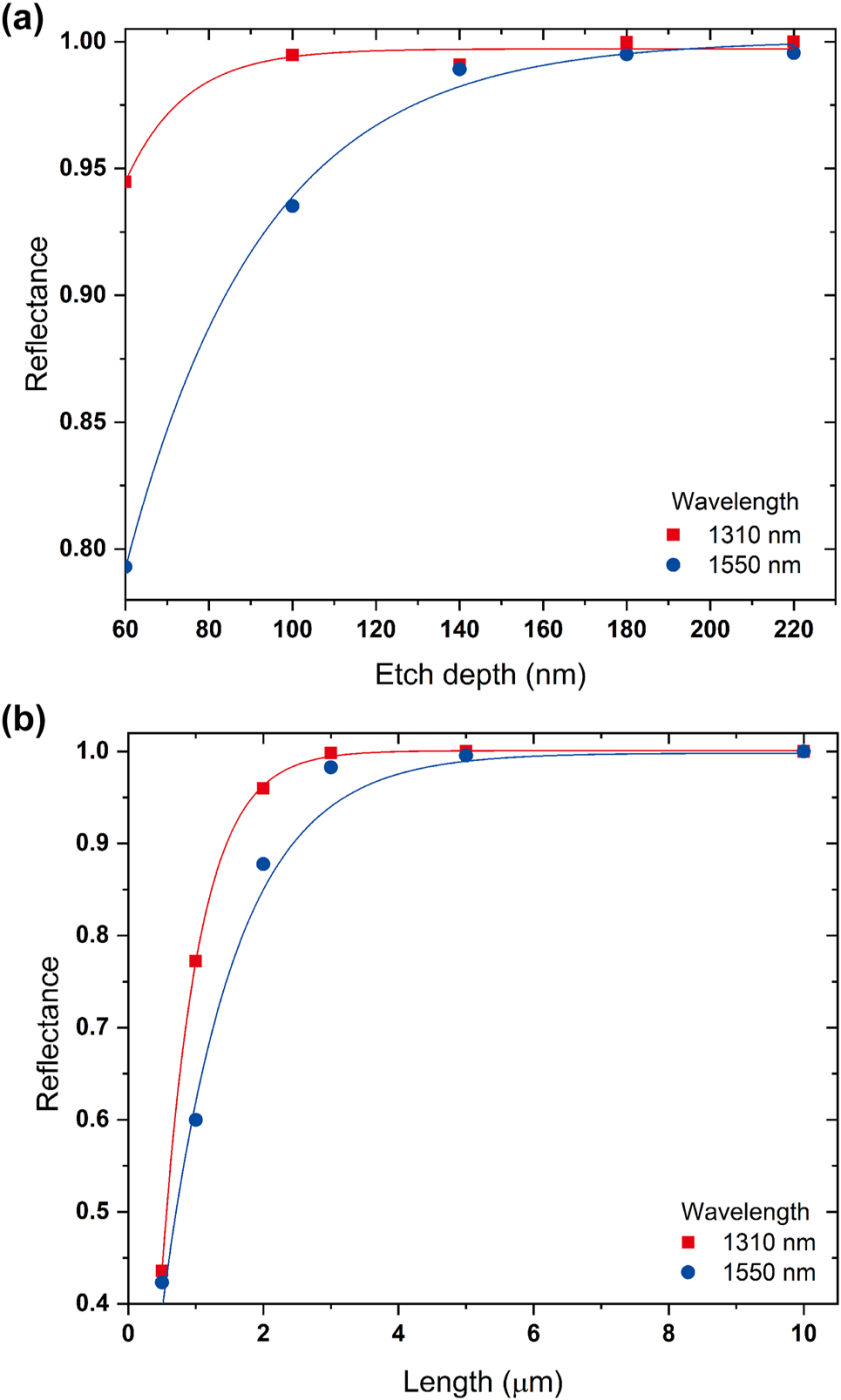
Relation between reflectance of the inversely designed reflector and geometric parameters. (a) Etch depth of Si. (b) Inverse design length.

The relation between reflectance of each reflector and geometric parameters is shown in Figure 4. In order to achieve high reflectance as 0.99, the minimum etch depth and inverse design length is 100 nm and 2.5 μm each. In case of the Reflector 2, the minimum etch depth is about 170 nm and the minimum length for the inverse design is estimated to be 4.5 μm.

We investigated the effect of the size of the nodes (d*x*) in the inverse design area, which represents the resolution of the inverse design. We conducted the identical inverse design process by reducing d*x* from 50 nm to 1 nm for *L* = 5 μm and *d* = 220 nm. When d*x* equals to 50 nm, the reflectance of the Reflector 1 is just about 0.723. However, the reflectance of the Reflector 1 becomes saturated when d*x* equals to 15 nm. In case of the Reflector 2, the reflectance is 0.68 when d*x* equals to 50 nm. Then it is also saturated when d*x* is about 10 nm. The reflectance of each reflector is shown in Supplementary Material as Figure S3.

For estimating the fabrication tolerance, we evaluated the reflectance of varied inverse design patterns, which have different partially etched Si area. We have changed the area of the partially etched Si area to 10 nm narrower or wider. We displayed the reflectance spectra of the Reflector 1 and 2 in Supplementary Material in Figure S4. For the Reflector 1, even when the area is wider or narrower, the change of the reflectance spectrum is not huge. When the area is wider, the reflectance at the wavelength of 1310 nm is 0.9992, and when the area is narrower, the reflectance is 0.991. Also, for the Reflector 2, when the area is wider, the reflectance at the wavelength of 1550 nm is 0.99. When the area is narrower, the reflectance is changed to 0.992. Therefore, the proposed inversely designed reflectors have strong fabrication tolerance properties.

## 3 Fabry–Perot resonators based on the inversely designed Si photonic waveguide reflectors

**Figure 5:**
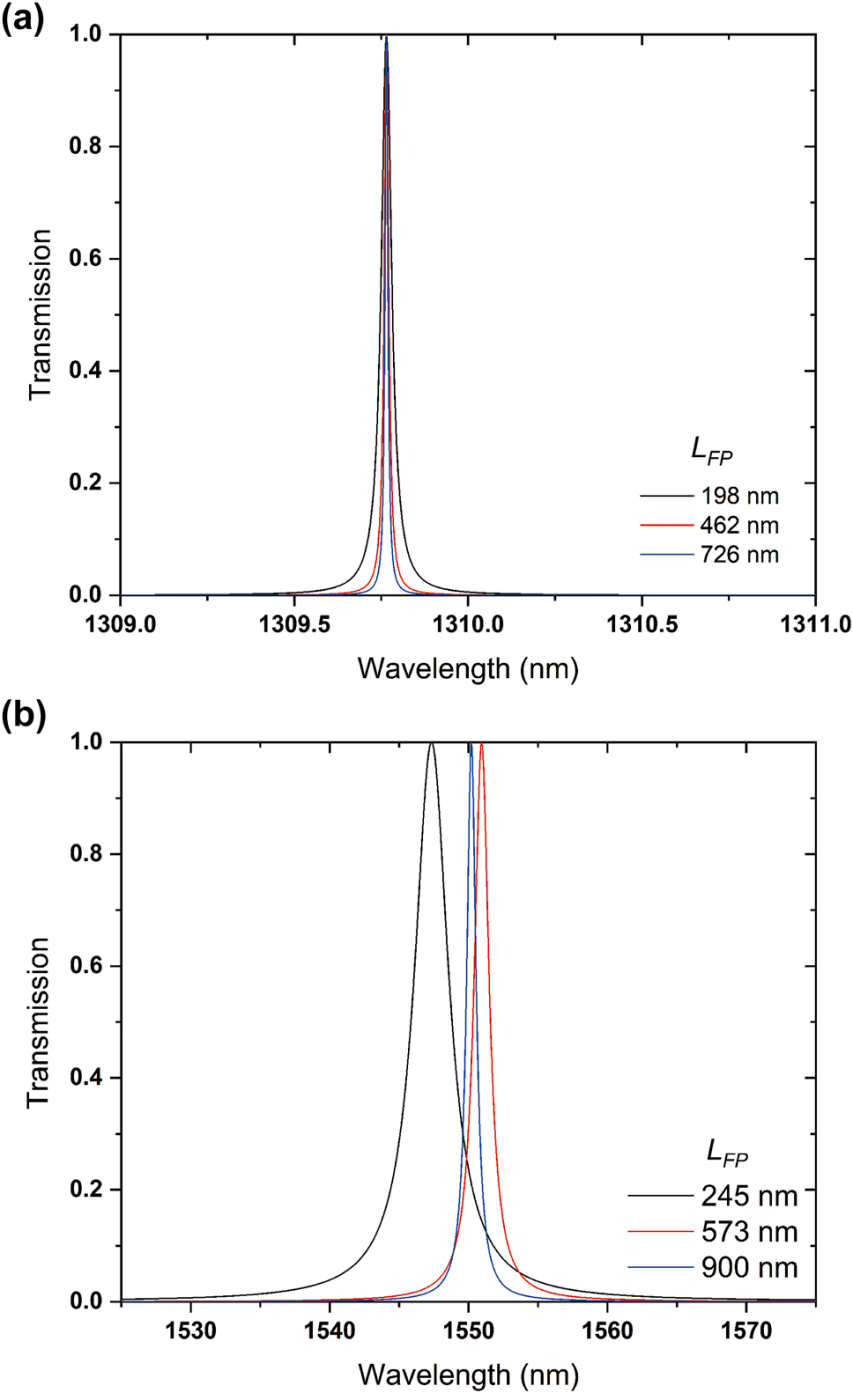
Transmission spectra of the Fabry–Perot resonators based on the inversely designed reflectors. (a) Reflector 1 and (b) Reflector 2 with *L* = 5 μm and *d* = 220 nm.

We investigated the Fabry–Perot resonator composed of two identical inversely designed photonic waveguide reflectors and a photonic waveguide between two reflectors, which is described in Figure 5. In order to determine the length of the photonic waveguide between two reflectors, the phase change from the reflector should be considered. In order to calculate the phase change from the inversely designed photonic waveguide reflectors, we simulate two structures, one is a single straight Si waveguide without any reflectors and the other is a Si waveguide with the reflector at the end of the waveguide. By comparing two structures, we can estimate the phase difference by the waveguide reflector. The length of the Fabry–Perot resonator, *L*_FP_, is determined by a condition that satisfies a constructive interference between the propagating wave and the reflected wave by the reflector [37]. The condition for the constructive interference is explained in Equation (3). *k*_*p*_ is the wavevector of the propagating wave, *n*_*g*_ is the group index of the propagating TE_0_ mode of the Si waveguide, and *λ* is the target wavelength.

(3a) $$2k_{p}L_{\text{FP}}+\Delta \phi =2m\pi ,$$

(3b) $$k_{p}=2\pi n_{g}/\lambda .$$

**Figure 6:**
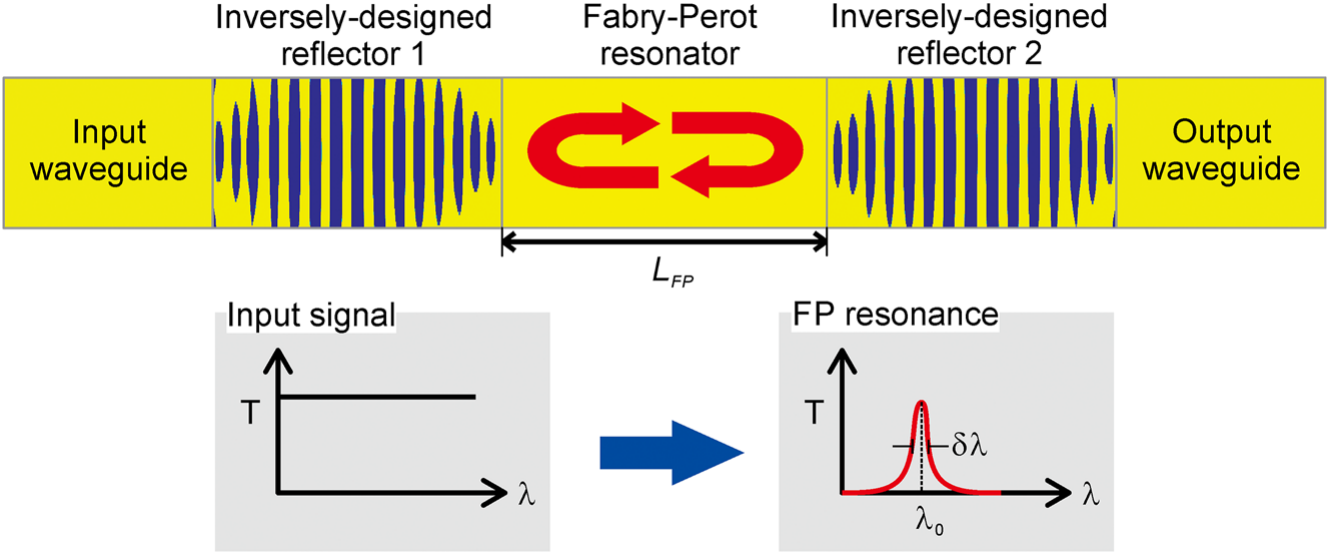
Device schematic of the Fabry–Perot resonator based on the inversely designed Si photonic waveguide reflectors.

In Equation (3), Δ*ϕ* is phase shift by the waveguide reflector and m is any positive integer. The calculation of the phase shifts is conducted by calculating phases of the reflected electromagnetic waves by the device, and the calculated phase shift is *π*/2 for each reflector. By Equation (3), we can calculate the length for the Fabry–Perot resonator for both the wavelength of 1310 nm and 1550 nm. In the Fabry–Perot resonators, the waveguide reflectors have been inversely designed with *d* = 220 nm and *L* = 5 μm. For the wavelength of 1310 nm, *L*_FP_ is calculated to be 198 nm, 462 nm, 726 nm … by increasing m, and for the wavelength of 1550 nm, *L*_FP_ is calculated to be 245 nm, 573 nm, 900 nm … by increasing m. In order to characterize the effect of the length on the Fabry–Perot resonators, we simulated the Fabry–Perot resonators with different *L*_FP_s by FDTD simulations for acquiring the transmittance spectra of the resonators, and they are shown in Figure 6. The transmittance spectra of the resonator whose target wavelength is 1310 nm are shown in Figure 6(a), and the spectra of the resonator for the wavelength of 1550 nm are shown in Figure 6(b). For the wavelength of 1310 nm, the resonant wavelengths for *L*_FP_ = 198 nm, 462 nm, and 726 nm are calculated to 1309.77 nm, 1309.76 nm, and 1309.76 nm, which is very similar to the estimated value from Equation (2). And, the optical bandwidths of the resonators for *L*_FP_ = 198 nm, 462 nm, and 726 nm are calculated to 0.035 nm, 0.014, nm and 0.01 nm. As the length of the resonator increases, the bandwidth of the resonators decreases as well. So, the quality factors (*Q* factors) of the resonators, which can be calculated by *λ*_0_/*δλ* where *λ*_0_ is resonant wavelength and *δλ* is the optical bandwidth, are 37,422, 93,555, and 130,980. However, if the length of the reflector is changed to 2 μm, which has a reflectance of 0.96 at the wavelength of 1310 nm, the optical bandwidths of the Fabry–Perot resonators become wider. When *L*_FP_ equals to 207 nm, 483 nm, and 760 nm, the optical bandwidths of the resonators are 22.59 nm, 9.65 nm, and 6.18 nm, which are shown in supplementary material, Figure S5. Those optical bandwidths of the resonators correspond to the *Q* factors of 57.87, 135.47, and 211.82.

## 4 Conclusions

We proposed the inverse designs of the Si waveguide reflectors and their applications as components for the Fabry–Perot resonators for the wavelength of 1310 nm and 1550 nm. The inversely designed waveguide reflectors have the reflectance of 0.99993 for the wavelength of 1310 nm and 0.9955 for the wavelength of 1550 nm with 5-μm-long device length. With these compact waveguide reflectors, we estimate the Fabry–Perot resonators, which are composed of two reflectors and a waveguide between reflectors. The length of the waveguide between reflectors have been analytically calculated and tested by FDTD simulation. The optical bandwidths of the resonators for the wavelength of 1310 nm and 1550 nm are calculated to be 0.01 nm and 0.6 nm each, and the *Q* factors of the resonators for the wavelength of 1310 nm and 1550 nm are estimated to be 130,980 and 2583. For comparison, we have compared the device footprints and *Q* factor of the waveguide resonant devices such as microring resonators, photonic waveguide cavities, and the Fabry–Perot resonator in this paper in Table 1.

**Table 1:** Comparison with other photonic waveguide cavities.

Types	Waveguide coupling	Device footprint	Quality factor	Reference
MRR	Directional coupler	30 × 30 μm^2^	57,000	[2]
PhC	Difficult	Few μm	260,000	[7]
FP resonators	Single waveguide	200 μm	19,000	[10]
In this work	Single waveguide	10.7 μm	130,000	

We expected that the Fabry–Perot resonators with the inversely designed waveguide reflectors can be utilized for the external cavity lasers, optical waveguide sensors, and optical modulators with very small device footprints.

## Supplementary Material

Supplementary Material Details
