# Supplementary material for: Inverse design of compact silicon photonic waveguide reflectors and their application for Fabry–Perot resonators
Source: Nanophotonics. 2024 Apr 16;13(15):2829–37. doi: 10.1515/nanoph-2024-0017 (PMC11501554; doi:10.1515/nanoph-2024-0017)
Supplement: Supplementary file 1 — Supplementary Material Details [file j_nanoph-2024-0017_suppl_001.pdf]

Supplementary Material

Inverse design of compact silicon photonic waveguide reflectors and their applications for Fabry-Perot resonators

Y. Kim and S.-H. Hong

Electronics and Telecommunications Research Institute

Comparison of the inversely-designed Si photonic waveguide reflectors with the Si photonic waveguide DBRs

In order to verify the inverse design of the Si photonic waveguide reflectors in the main text, we conducted FDTD simulation about DBRs based on the Si photonic waveguides. The DBRs have identical geometric parameters (etch depth of Si and the length of design area). Fig. S1(a) and S1(b) show the reflectance spectra of the DBRs depending on the etch depth of Si for the wavelength of 1310 nm and 1550 nm each. The length of the DBRs for Fig. S1 is set to 5 μm. And, the period and duty cycle of the DBR are determined by the conventional design method for DBRs [S1].

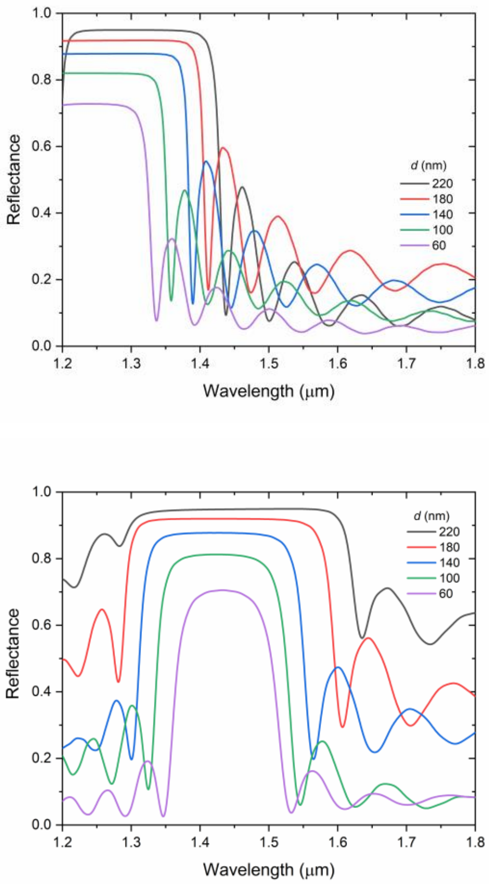

Fig. S1. Reflectance spectra of the DBR for the target wavelength of (upper) 1310 nm and (lower) 1550 nm depending on the etch depth of Si

Fig. S2(a) and S2(b) show the reflectance spectra of the DBRs depending on the length of the DBRs for the wavelength of 1310 nm and 1550 nm each.

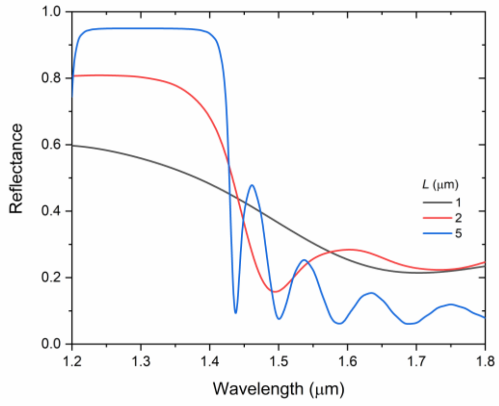

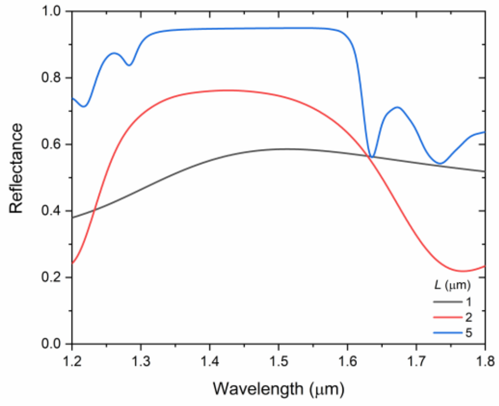

Fig. S2. Reflectance spectra of the DBR for the target wavelength of (upper) 1310 nm and (lower) 1550 nm depending on the length of DBRs

Effect of the size of the nodes in the inverse design area

We investigated the effect of the size of the nodes in the inverse design area (*dx*) for each reflector by analyzing the reflectance at the target wavelength for reducing the size of the nodes from 50 nm to 1 nm. And, Fig. S3 shows the reflectance value as a function of *dx* for each reflector. As mentioned in the main text, for both reflectors, *dx* = 10 nm gives the saturated reflectance. So, we decided the value of the *dx* is 10 nm for computational time efficiency.

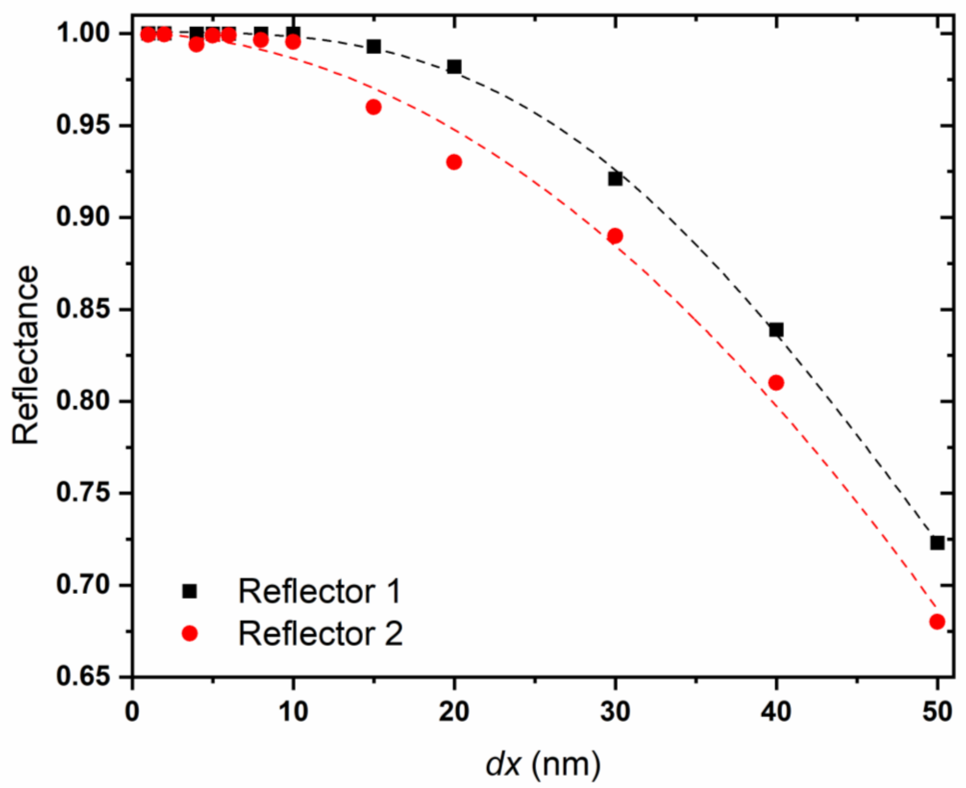

Fig. S3. Reflectance as a function of *dx* for Reflectance 1 and Reflector 2. Reflectance of each reflector is at the target wavelengths.

Fabrication tolerance property of the photonic waveguide reflectors

Also, we investigated the fabrication tolerance property by a method mentioned in the main text. The reflectance spectra for three cases (+10 nm, original, and -10 nm) are shown in Fig. S4. As mentioned in the main text, the fabrication error about 10 nm does not affect the reflectance spectrum critically.

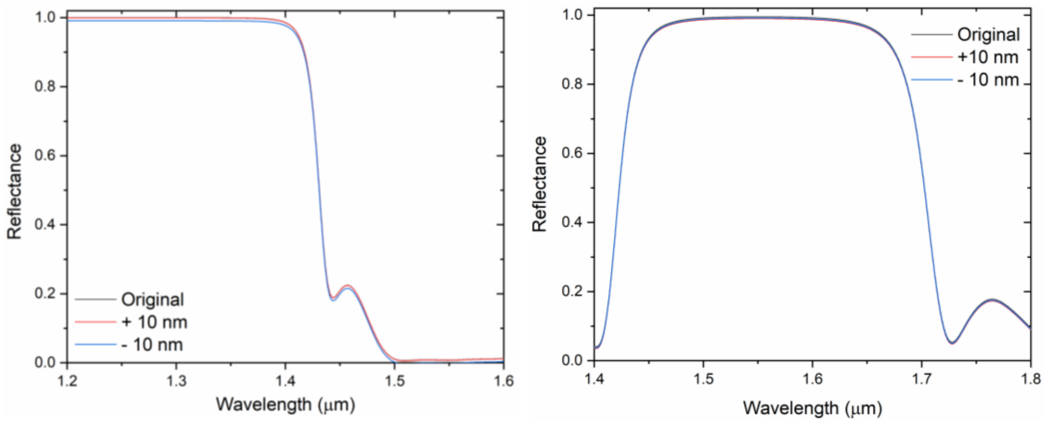

Fig. S4. Reflectance spectra for Reflector 1 (left) and Reflector 2 (right) when the error in partially-etched Si area occurs. +10 nm indicates the etched Si area becomes wider and -10 nm indicates the etched Si area becomes narrower.

Fabry-Perot resonators based on the reflectors which have lower reflectance

In order to verify the Fabry-Perot resonators based on the inversely-designed photonic waveguide reflector in the main text, we simulated the Fabry-Perot resonators with lower reflectance reflectors. We reduced the length of the photonic waveguide reflectors for both target wavelengths of 1310 nm and 1550 nm from 5-µm to 2 µm for the wavelength of 1310 nm and 4 µm for the wavelength of 1550 nm. In case of 2 µm long photonic waveguide reflector for the wavelength of 1310 nm, it has a reflectance of 0.96. And, in case of 4 µm long photonic waveguide reflector for the wavelength of 1550 nm, it has a reflectance of 0.987. Using these photonic waveguide reflectors, we designed the Fabry-Perot resonators with different Fabry-Perot cavity lengths (L_{FP} in main text). L_{FP} for the wavelength of 1310 nm with the 2-µm-long reflectors is calculated to 207 nm, 483 nm, 760 nm and so on by increasing the order m in the main text. L_{FP} for the wavelength of 1550 nm with the 4-µm-long reflectors is calculated to 234 nm, 547 nm, 859 nm and so on by increasing the order m.

The simulated resonant wavelengths for the wavelength of 1310 nm are estimated to 1307.37 nm, 1307.27 nm, and 1309.08 nm by increasing m. And optical bandwidths are 22.59 nm, 9.65 nm, and 6.18 nm by increasing m.

Therefore, Q factors of the resonator for the wavelength of 1310 nm are calculated to 57.8695, 135.4665, and 211.8241.

In case of the simulation results about the resonators for the wavelength of 1550 nm, the simulated resonant wavelengths are estimated to 1548.13 nm, 1545.11 nm, and 1542.45 nm by increasing *m*. And, the optical bandwidths of the resonators are 8.58 nm, 3.66 nm, and 2.32 nm by increasing *m*. So, the Q factors of the resonators for the wavelength of 1550 nm are` calculated to 180.4333, 422.1606, and 664.8488.

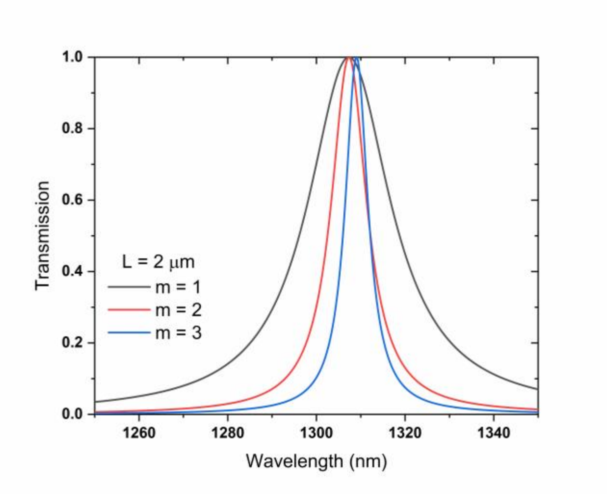

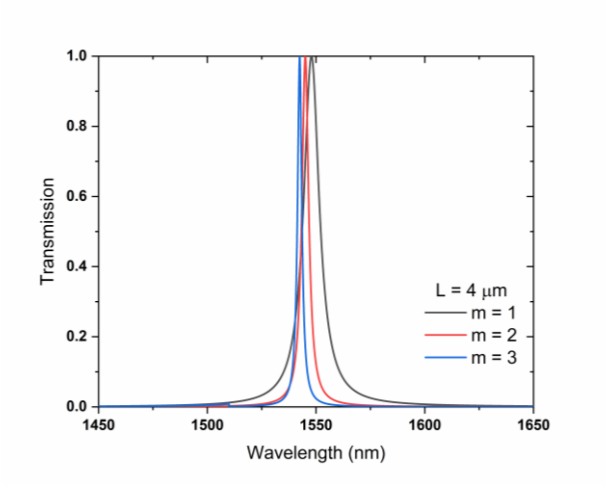

Fig. S3 Transmission spectra of the Fabry-Perot resonators with lower Q factors (Upper) FP resonators with 2-μm-long reflectors for the wavelength of 1310 nm (Lower) FP resonators with 4-μm-long reflectors for the wavelength of 1550 nm

Reference

[S1] A. Yariv and P. Yeh, "Wave propagation in periodic media," in *Photonics*: *Optical Electronics in Modern Communications*, 6th ed., Oxford, England: Oxford University Press, 2007, pp. 539-601.
